# A case of Fournier’s gangrene in a young immunocompetent male patient resulting from a delayed diagnosis of appendicitis

**DOI:** 10.1093/jscr/rjw058

**Published:** 2016-04-22

**Authors:** Michael Wanis, Shady Nafie, John Kilian Mellon

**Affiliations:** Department of Urology, Leicester General Hospital, University Hospitals of Leicester NHS Trust, Gwendolen Road, Leicester, LE5 4PW, UK

## Abstract

We discuss the case of a 28-year-old male patient presenting to our department with an atypical history of acute scrotal swelling on a background of abdominal pain. He was diagnosed with a perforated appendicitis and Fournier’s gangrene.

## Introduction

By definition, Fournier’s gangrene is a synergetic polymicrobial necrotising fasciitis of the perineal, perianal and genital areas. It is a rare condition that can occur in both males and females with a male preponderance of 10:1. It is potentially life-threatening with a mortality rate of up to 40%. There is a higher incidence in diabetic and immunocompromised patients and it is usually associated with a history of trauma, urinary tract infection or perirectal infection [1].

We discuss a case of a male patient presenting to our department with an atypical history of acute scrotal swelling on a background of abdominal pain, who was diagnosed with Fournier’s gangrene secondary to a delayed diagnosis of perforated appendicitis.

## Case Presentation

A 28-year-old man was admitted to the Urology department via the emergency department with a 24-hour history of acute, rapidly progressing bilateral scrotal swelling, pain and erythema.

He reported lifting a heavy object at work 10 days prior to this, resulting in the sudden onset of right iliac fossa pain, radiating to the groin. Three days later he felt feverish and developed flu-like symptoms, which gradually worsened over the forthcoming week. He was diagnosed with ‘muscle strain’ by the out-of-hours service, having been clinically well with normal vital signs at the time.

On hospital admission, he appeared unwell with facial flushing but was apyrexial. On examination, he was mildly tachycardic but normotensive. There was right iliac fossa and right renal angle tenderness with signs of peritonism along with diffuse swelling, erythema and tenderness. Digital rectal examination revealed no evidence of a perianal abscess or prostatic tenderness. His blood results revealed an elevated White Cell Count of 33 × 10^9^/l and CRP of 493 mg/l in addition to an acute kidney injury with an eGFR of 43 ml/min and creatinine of 172 umol/l.

**Figure 1:**
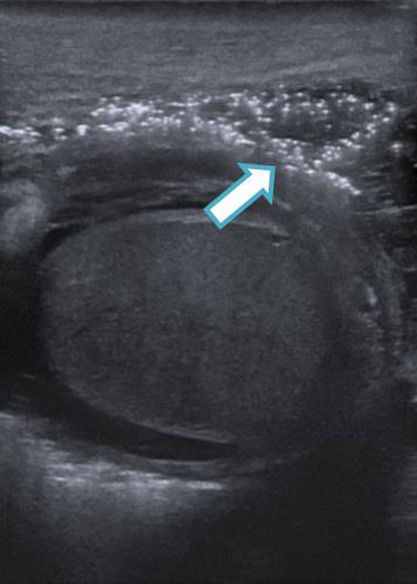
Ultrasound image demonstrating gas within the scrotum.

**Figure 2:**
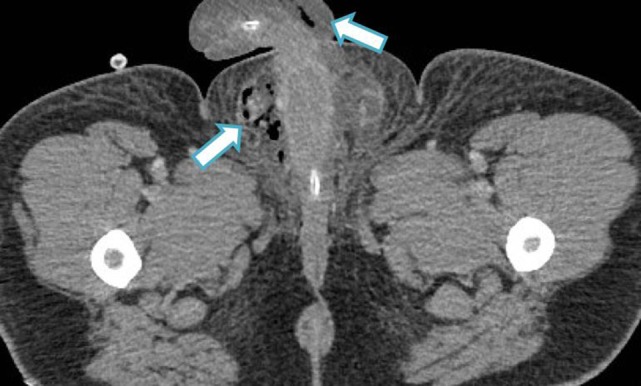
CT image (axial slice) showing gas within the scrotum and penile shaft.

**Figure 3:**
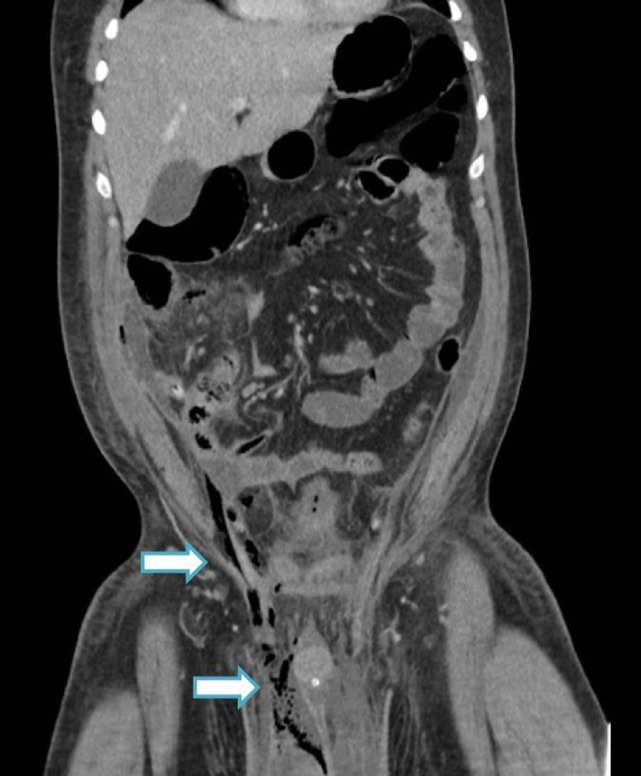
CT image (coronal slice) showing gas tracking from the right iliac fossa, down the spermatic cord into the scrotum.

An urgent scrotal ultrasound demonstrated evidence of gas within the deep tissues of the scrotum but no abscess as shown in Fig. 1. Subsequently an abdomino-pelvic CT scan revealed multi-loculated collections in the right iliac fossa, suggesting perforated appendicitis with secondary extension of gas down the spermatic cord into the scrotum, as shown in Figs 2 and 3.

A urethral catheter was inserted for urinary diversion and intravenous piperacillin-tazobactam and clindamycin were commenced, as per microbiology advice. The patient underwent an urgent laparotomy by the general surgical team. Washout and drainage of the right iliac fossa collection were performed. The appendix was absent, having disintegrated within the abscess. Post-operatively he was admitted to the Intensive Care Unit (ICU) for cardiovascular support and sepsis management. Fourty eight  hours later, there was evidence of spreading cellulitis across the lower abdomen as well as focal areas of ischaemia on the scrotal skin, prompting a second visit to the operating theatre for emergency debridement. This revealed evidence of gross underlying soft tissue necrosis extending deep into the scrotum, perineum and lower abdomen. A wound inspection took place 48 hours later revealing healthy tissues and no further debridement was necessary. The patient was successfully extubated four days later, and discharged from ICU and general surgical care. Subsequently, he made a good recovery and was discharged home after two weeks, with the involvement of the plastic surgery team to follow up his wound as an outpatient.

## Discussion

Fournier’s gangrene is a rare life-threatening condition that typically occurs in elderly, immunocompromised and diabetic patients. Outside this typical cohort of patients, a diagnosis of Fournier’s gangrene may be overlooked. On the other hand, acute appendicitis is a relatively common condition that predominantly occurs in the 15- to 59-year age range and has a variable presentation. A delayed or missed diagnosis could lead to serious complications.

There are cases in the literature of acute appendicitis, in particular retrocaecal, or diverticulitis complicated by necrotiing fasciitis, most commonly occurring in older patients with medical comorbidities [2–6]. Progression to necrotising fasciitis may result from a delayed diagnosis or conservative management, and consequently the development of intra-abdominal abscesses. A diagnosis of Fournier’s gangrene may not be clear initially if the underlying source is intra-abdominal, thus resulting in a late presentation and a poorer prognosis [4].

Our case illustrates the importance of considering a diagnosis of Fournier’s gangrene even if the patient is young, fit and immunocompetent, as the consequences of missing this diagnosis can be catastrophic. Furthermore, when there is a clinical suspicion of Fournier’s gangrene and the underlying aetiology is unclear, after exclusion of trauma, urinary tract and perirectal infection, an intra-abdominal cause should be considered.

Fournier’s gangrene is a rare but potentially life-threatening complication of acute appendicitis that could occur in patients of any age, and notably, should not be overlooked in young, healthy patients. A high index of suspicion as well as timely surgical management and a multidisciplinary approach are necessary in order to maximise the chances of a favourable outcome.
